# Mycotoxin Exposure and Renal Cell Carcinoma Risk: An Association Study in the EPIC European Cohort

**DOI:** 10.3390/nu14173581

**Published:** 2022-08-30

**Authors:** Liesel Claeys, Sarah De Saeger, Ghislaine Scelo, Carine Biessy, Corinne Casagrande, Genevieve Nicolas, Michael Korenjak, Beatrice Fervers, Alicia K. Heath, Vittorio Krogh, Leila Luján-Barroso, Jesús Castilla, Börje Ljungberg, Miguel Rodriguez-Barranco, Ulrika Ericson, Carmen Santiuste, Alberto Catalano, Kim Overvad, Magritt Brustad, Marc J. Gunter, Jiri Zavadil, Marthe De Boevre, Inge Huybrechts

**Affiliations:** 1 Centre of Excellence in Mycotoxicology and Public Health, Department of Bioanalysis, Ghent University, Ottergemsesteenweg 460, 9000 Ghent, Belgium; 2 Cancer Research Institute Ghent (CRIG), 9000 Ghent, Belgium; 3 Epigenomics and Mechanisms Branch, International Agency for Research on Cancer, World Health Organization, 150 Cours Albert Thomas, 69008 Lyon, France; 4 Nutrition and Metabolism Branch, International Agency for Research on Cancer, World Health Organization, 150 Cours Albert Thomas, 69008 Lyon, France; 5 Department of Biotechnology and Food Technology, Faculty of Science, University of Johannesburg, Doornfontein Campus, Johannesburg 2092, South Africa; 6 Genomic Epidemiology Branch, International Agency for Research on Cancer, World Health Organization, 150 Cours Albert Thomas, 69008 Lyon, France; 7 Department Prevention Cancer Environment, Centre Léon Bérard, U1296 INSERM Radiation, Defense, Health and Environment, 28 Rue Laënnec, 69373 Lyon, France; 8 Department of Epidemiology and Biostatistics, School of Public Health, Imperial College London, St Mary’s Campus, Norfolk Place, London W2 1PG, UK; 9 Epidemiology and Prevention Unit, Fondazione IRCCS Istituto dei Tumori di Milano, 1 Via Venezian, 20133 Milan, Italy; 10 Unit of Nutrition and Cancer, Cancer Epidemiology Research Program, Catalan Institute of Oncology—IDIBELL, Granvia de L-Hospitalet 199-203, 08908 L’Hospitalet de Llobregat, Spain; 11 Navarra Public Health Institute—IdiSNA, Leyre 15, 31003 Pamplona, Spain; 12 Centre for Biomedical Research in Epidemiology and Public Health (CIBERESP), C. Monforte de Lemos 3-5, 28029 Madrid, Spain; 13 Department of Surgical and Perioperative Sciences, Urology and Andrology, Umeå University, SE-901 87 Umeå, Sweden; 14 Andalusian School of Public Health (EASP), 4 Cta. del Observatorio, 18011 Granada, Spain; 15 Instituto de Investigación Biosanitaria ibs. Granada, 15 Av. de Madrid, 18012 Granada, Spain; 16 Department of Clinical Sciences in Malmö, Lund University, Jan Waldenströms gata 35, SE-214 28 Malmö, Sweden; 17 Department of Epidemiology, Murcia Regional Heath Council, IMIB-Arrixaca, 11 Ronda de Levante, 30008 Murcia, Spain; 18 Department of Clinical and Biological Sciences, University of Turin, Regione Gonzole 10, 10143 Orbassano, Italy; 19 Department of Public Health, Aarhus University, Bartholins Allé 2, 8000 Aarhus, Denmark; 20 Department of Community Medicine, The Arctic University of Norway, Hansines veg 18, 9019 Tromsø, Norway

**Keywords:** mycotoxins, epidemiology, prevention, renal cancer, kidney cancer, Europe, exposure, external assessment

## Abstract

Background: Mycotoxins have been suggested to contribute to a spectrum of adverse health effects in humans, including at low concentrations. The recognition of these food contaminants being carcinogenic, as co-occurring rather than as singularly present, has emerged from recent research. The aim of this study was to assess the potential associations of single and multiple mycotoxin exposures with renal cell carcinoma risk in the European Prospective Investigation into Cancer and Nutrition (EPIC) cohort. Methods: Food questionnaire data from the EPIC cohort were matched to mycotoxin food occurrence data compiled by the European Food Safety Authority (EFSA) from European Member States to assess long-term dietary mycotoxin exposures, and to associate these with the risk of renal cell carcinoma (RCC, *n* = 911 cases) in 450,112 EPIC participants. Potential confounding factors were taken into account. Analyses were conducted using Cox’s proportional hazards regression models to compute hazard ratios (HRs) and 95% confidence intervals (95% CIs) with mycotoxin exposures expressed as µg/kg body weight/day. Results: Demographic characteristics differed between the RCC cases and non-cases for body mass index, age, alcohol intake at recruitment, and other dietary factors. In addition, the mycotoxin exposure distributions showed that a large proportion of the EPIC population was exposed to some of the main mycotoxins present in European foods such as deoxynivalenol (DON) and derivatives, fumonisins, *Fusarium* toxins, *Alternaria* toxins, and total mycotoxins. Nevertheless, no statistically significant associations were observed between the studied mycotoxins and mycotoxin groups, and the risk of RCC development. Conclusions: These results show an absence of statistically significant associations between long-term dietary mycotoxin exposures and RCC risk. However, these results need to be validated in other cohorts and preferably using repeated dietary exposure measurements. In addition, more occurrence data of, e.g., citrinin and fumonisins in different food commodities and countries in the EFSA database are a prerequisite to establish a greater degree of certainty.

## 1. Introduction

Renal cancers comprise malignant tumors of the renal parenchyma and renal pelvis. Adenocarcinoma of the renal parenchyma (referred to as renal cell carcinoma (RCC)) accounts for over 90% of renal cancers, while nearly all cancers arising in the renal pelvis are of the transitional cell type and comprise less than 10% of renal cancers [1,2]. The incidence rates of RCC have been increasing over the past decades, particularly in Western populations [3,4,5]. Mycotoxins were included as possible contributing factors in the development of Balkan endemic nephropathy (BEN), a unique chronic renal disease, alongside aristolochic acid (the principal etiologic agent for BEN recognized today), metals, metalloids, and other nephrotoxins [6,7,8].

Mycotoxins are toxic secondary metabolites produced by fungi that contaminate various agricultural commodities either before harvest, at harvest, or under post-harvest conditions [9]. A high number of filamentous fungi have the capability to produce mycotoxins; however, the most important producing genera are *Aspergillus*, *Fusarium*, and *Penicillium*. These fungi have a worldwide geographical distribution [10,11]. The main mycotoxins produced by the previously listed fungi are aflatoxins (AFs) and ochratoxin A (OTA), trichothecenes (e.g., T-2 toxin (T-2) and deoxynivalenol (DON)), zearalenone (ZEN), fumonisins (FBs), citrinin (CIT), and patulin (PAT) [12]. The toxins are potentially present in a wide range of vegetable products, in processed foods, beverages, feed, and animal products [13]. In terms of physicochemical properties, they are stable to a point where they cannot be completely destroyed during the different food processing procedures. Consuming contaminated foods leads to exposure to mycotoxins, which consequently enter the human body [12]. The International Agency for Research on Cancer (IARC) classifies mycotoxins according to the evidence of carcinogenicity to humans. All aflatoxins, including aflatoxin B1 (AFB1), are carcinogenic to humans, having the ability to cause extra-hepatic and hepatic carcinogenesis; therefore, they are categorized as Group 1 [12,14,15].

Natural nephrotoxicants within mycotoxins that possibly cause renal failure are OTA, FB1, and CIT [16]. OTA is suggested to have a carcinogenic effect on the kidneys, and CIT is one of the strongest nephrotoxins to animals [12]. Co-exposure to CIT and OTA is reflected in antagonism or synergy on affecting the kidneys [8]. FB1 was shown to be involved in the development of renal carcinomas in male rats [12,17]. A synergistic pattern could also be observed with OTA and FB1 at low concentrations, shifting to antagonism at high concentration levels [18]. In addition to the focus on the kidney, synergistic effects between ZEN and DON exposure are observed in HepG2 cells [19]. Nevertheless, there is a lack of studies investigating the impact of mycotoxin exposures on kidney cancer risk.

The goal of this study was to examine the associations of single and multiple mycotoxin exposures with the risk of developing RCC within the European Prospective Investigation into Cancer and Nutrition (EPIC), as a basis to develop future public health strategies.

## 2. Materials and Methods

### 2.1. Participants and Study Design

EPIC is a multicenter prospective cohort study designed to examine the relationship between lifestyle and cancer in Europe, including diet. The cohort includes 521,324 participants (mostly aged 35–70 years) in 23 centers located in 10 European countries, namely, Spain, Italy, France, Greece, Germany, the Netherlands, United Kingdom, Denmark, Sweden, and Norway. Enrolment, with a signed informed consent from each participant, took place between 1992 and 2000 at the different centers [20]. The rationale, study population, and data collection were described by Riboli et al. (1997) [21].

### 2.2. Exclusion Criteria

Participants with a prevalent cancer at any site at cohort entry (*n* = 25,184) and those with missing follow-up (*n* = 4128) or date of renal cancer diagnosis (*n* = 20) were excluded. In addition, participants from Greece were excluded (*n* = 26,915). Participants who did not complete the dietary or non-dietary baseline questionnaires (*n* = 5900), who withdrew from the study (*n* = 1), or who were in the top or bottom 1% of the ratio of energy intake to estimated energy requirement calculated from body weight, height, and age (*n* = 9064), were also excluded to reduce the impact on the analysis of implausible extreme values. In total, 71,212 participants were excluded. The final number of EPIC cohort participants available for these analyses was 450,112 (318,686 women and 131,426 men).

### 2.3. Assessment of Endpoints

Incident cancer cases were identified through population cancer registries (Denmark, Italy, the Netherlands, Spain, Sweden, the United Kingdom, and Norway) or through active follow-up (France and Germany), depending on the follow-up system in each of the participating centers. Active follow-up used a combination of methods, including health insurance records, cancer and pathology registries, and direct contact with participants or their next of kin. Participants were followed from study entry until cancer diagnosis, death, emigration, or end of follow-up period. Mortality data were coded following the rules of the 10th revision of the International Statistical Classification of Diseases, Injuries, and Causes of Death (ICD-10) and cancer incidence data following the third revision of the International Classification of Diseases for Oncology (ICD-O-3). Data were coded according to ICD-10/ICD-O-3 as carcinoma of the renal parenchyma (C64.9) and carcinoma of the renal pelvis (C65.9), sarcoma, and unclear or inconsistent subsite (including incoherence between topography and histological codes, missing or vague histological code, inconsistency between the level of details and the source of information). In this study, RCC (C64.9) was considered as the outcome of interest.

### 2.4. Dietary Data and Lifestyle Questionnaires

Information on physical activity, history of tobacco smoking, alcohol consumption, and education was collected at baseline by questionnaires. Weight and height were measured at baseline in all centers, except from parts of Oxford (UK), France, and Norway, where weight and height were self-reported [20].

#### 2.4.1. Dietary Questionnaires

Usual dietary intakes were assessed at study baseline using validated country/center-specific dietary questionnaires (DQs). In most centers, DQs were self-administered, with the exception of Ragusa (Italy), Naples (Italy), and Spain, where face-to-face interviews were performed. Extensive quantitative DQs were used in northern Italy, the Netherlands, and Germany and were structured by meals in Spain, France, and Ragusa. Semi-quantitative food-frequency questionnaires (FFQs) were used in Denmark, Norway, Naples, and Umeå (Sweden). In the United Kingdom, both a semi-quantitative FFQ and a 7 day record were used, whereas a method combining a short non-quantitative FFQ with a 7 day record on hot meals was used in Malmö (Sweden) [20]. The latter was a 168-item questionnaire where portion sizes were indicated using a picture book with photos on four different portion sizes [22,23].

To prepare the EPIC Nutrient Database (ENDB) for the EPIC study, a highly standardized procedure was used, adopting nutrient values from 10 national food composition databases of the respective EPIC countries. The process for compiling this ENDB database was previously described by Slimani et al. (2007) [24].

#### 2.4.2. Mycotoxin Occurrence Data

For this study, mycotoxin occurrence data obtained through the European Food Safety Authority (EFSA) were used and matched with the EPIC food consumption data derived from the dietary questionnaires. The EFSA database relevant to this project records the clustered mycotoxin occurrences of all types of mycotoxins, filed in Europe and obtained via the European Member States. To calculate the quantity of each mycotoxin consumed by a specific individual, the portion (in grams) of every food that was consumed by each individual (as reported in the FFQs) was linked to the mycotoxin occurrence data for that particular food.

#### 2.4.3. Concentration Scenarios Regarding Mycotoxin Concentrations

When reporting contaminant concentrations analyzed in monitoring programs, actual numeric values of concentrations are only reported when the measurements exceed the limit of detection (LOD) or limit of quantification (LOQ). In these exposure assessments performed within EPIC, a middle-bound (MB) concentration scenario was built by assigning a concentration equal to half the limit (LOD or LOQ) value when the concentration value was missing or below the LOD or LOQ. When all the concentrations of a mycotoxin were missing, the food was assumed to contain no mycotoxins. This scenario was chosen as a more optimal approach as opposed to assigning a concentration equal to 0 µg/kg when the concentration value was missing (so called lower-bound scenario). However, the lower-bound scenario was also used in this study for conducting analyses. This end-user mycotoxin database was further applied to investigate single and multiple mycotoxin exposures in the EPIC cohort and its association with RCC risk.

#### 2.4.4. Mycotoxin Grouping for Analysis

Groups of related mycotoxins were computed by summing the levels of mycotoxins belonging to certain families depending on their chemical structure. The group *Aflatoxins* included AFB1, AFB2, AFG1, AFG2, and AFM1. The group of *Deoxynivalenol and derivatives* included DON, 3-acetyl-DON (3ADON), 15-acetyl-DON (15ADON), and deoxynivalenol-3-glucoside (D3G). The *Fumonisins group* included FB1, FB2, and FB3. *Zearalenone and derivatives* constituted the sum of ZEN, ZEN-derivatives, sum of zearalenols (ZEL), α-zearalenol (α-ZEL), β-zearalenol (β-ZEL), and zearalanone (ZAN). The *Alternaria toxins group* included alternariol (AOH), alternariol methylether (AME), altenuene (ALT), tenuazonic acid (TEA), altertoxin (ATX), tentoxin (TEN), and AAL toxins (AAL_toxins). The *Enniatins group* included enniatin A (ENA), enniatin A1 (ENA1), enniatin B (ENB), and enniatin B1 (ENB1). The *Ergot alkaloids group* included ergocornine (Eco), ergocorninine (Econ), ergocristine (Ecr), ergocristinine (Ecrn), α-ergokryptine (Ek), α-ergokryptinine (Ekn), ergometrine (Em), ergometrinine (Emn), ergosine (Es), ergosinine (Esn), ergotamine (Et), and ergotaminine (Etn). The *Ochratoxins* group included OTA. The *group T2 and HT2* included HT-2 toxin (HT2) and T-2 toxin (T2). Other mycotoxins were handled individually, namely, PAT, nivalenol (NIV), diacetoxyscirpenol (DAS), fusarenon-X (FUS-X), sterigmatocystin (STC), moniliformine (MON), citrinin (CIT), and beauvericin (BEA). The *Fusarium toxins group* consisted of the sum of ‘Deoxynivalenol and derivates’, ‘group T2 & HT2′, ‘Fumonisins group’, ‘Zearalenone and derivatives’, NIV, and DAS.

### 2.5. Statistical Analysis

Analyses were based on mycotoxin intakes (in µg/day) divided by kilograms of body weight (bw), expressed as an exposure estimate in µg/kg bw/day. In addition to the total multi-mycotoxin exposures, groups of mycotoxins were computed by summing the levels of mycotoxins belonging to certain families depending on their chemical structure, as reflected in Section 2.4.4.

Descriptive analyses were conducted to investigate the difference between RCC cases and non-cases, reporting the mean and standard deviation (SD) for continuous variables and percentages for categorical variables. Mycotoxin distributions were analyzed, and the following parameters were presented: minimum, maximum, and percentiles (P1, P5, P10, P25, P50, P75, P90, P95, and P99).

Cox’s proportional hazards regression using age as the underlying time metric with the participants’ age at recruitment as the entry time and their age at cancer diagnosis, death, emigration, or last complete follow-up, whichever occurred first, as the exit time was used to estimate the hazard ratio (HR) and 95% confidence interval (CI) for the association between dietary mycotoxin exposure and RCC risk. Mycotoxin levels were log_2_-transformed (to account for a doubling of the continuous exposure) and divided into sex-specific tertiles on the basis of their distribution in all cohort participants at baseline, setting participants in the lowest category of mycotoxin exposure as the reference group. All models were stratified by sex, study center, and age at enrolment. Multivariable models were adjusted for known or suspected risk factors for RCC according to the findings of the World Cancer Research Fund/American Institute for Cancer Research [25]. Lastly, confounding factors, according to the literature, remained in the models if the β-estimate changed by more than 10. The final multivariable model included body mass index (BMI), education level (none, primary school completed, technical/professional school, secondary school, longer education (including university degree), or not specified), Cambridge physical activity index (inactive, moderately inactive, moderately active, active, or missing), diabetes (no, yes, or do not know), hypertension (no, yes, or do not know), smoking status (never, former, smoker, or unknown), alcohol intake at recruitment, and energy intake. Tests for trends in HRs by tertiles were computed by assigning consecutive scores to the tertiles.

Statistical analyses were performed with SAS statistical software package (version 9.4). All tests of statistical significance were two-sided, and *p*-values below 0.05 were considered significant.

## 3. Results

After a median follow-up of 14.9 years, 911 incident cases of RCC were reported among the 450,112 participants. Demographic characteristics differed between the RCC cases and non-cases for BMI, sex, age, alcohol at recruitment, and other lifestyle and risk factors (Table 1). Compared with non-cases, RCC cases were more likely to be older, be men, have a higher BMI, drink more alcohol, have lower educational attainment, be current smokers, and have diabetes or hypertension.

Table 2 describes the external mycotoxin exposures assessed on the basis of dietary questionnaire data for the EPIC cohort in the lower- and middle-bound scenarios. The mycotoxin exposure distributions (Table 2 and Table S1) showed that a large part of the EPIC population was exposed to some of the main mycotoxins present in European foods such as *DON and derivatives*, *fumonisins*, *Fusarium* toxins, *Alternaria* toxins, and total mycotoxins. The estimated median total mycotoxin exposure was 0.86 (interquartile range 0.62–1.16) µg/kg body weight per day (for the middle-bound scenario) in non-cases. Additionally, the group of *Fusarium* toxins made up a large portion of total mycotoxin exposure, with a median of 0.55 (interquartile range 0.40–0.74) µg/kg body weight per day in non-cases. Thus, the estimated external mycotoxin exposures for the EPIC population were lower than the safety reference values set by EFSA (Table S2).

Cox hazard regressions were used to investigate the possible association between mycotoxin exposures and RCC risk. Tests for trends in HRs by tertiles were computed by assigning consecutive scores to the tertiles. No *p*-values between the tertiles, known as the TrendTest, and no *p*-values comparing each tertile with the reference, known as ProbChiSq, were below 0.05. (Table 3) Therefore, no statistically significant association could be assigned between a studied mycotoxin or mycotoxin group and risk of RCC development. Moreover, the multi-adjusted Cox hazard models did not result in significant associations between the individual mycotoxin exposures and RCC risk. The HR for the first and third tertiles of total mycotoxin exposure was 0.81 (95% CI 0.62–1.07).

## 4. Discussion

### 4.1. Mycotoxin Exposure Distribution and Association with RCC Risk

In this cohort study of participants from nine European countries, we found that a large proportion of the EPIC population was likely exposed to some of the main mycotoxins present in European foods, such as *DON and derivatives*, *fumonisins*, *Fusarium* toxins, *Alternaria* toxins, and total mycotoxins (Table 2). However, none of the individual mycotoxins or mycotoxin groups were associated with risk of RCC.

*Deoyxnivalenol and derivatives* are mainly found in cereals and cereal-based products [26]. *Fumonisins* are almost exclusively found in maize [27]. Other mycotoxins included in *Fusarium toxins* are mainly found in oats, maize, barley, wheat, and cereal grains [28,29,30,31]. Lastly, the *Alternaria toxins* contaminate cereals, oilseeds, fruits and vegetables [32]. These results are in line with the main crops consumed in Europe [33].

When comparing the estimated external mycotoxin exposure to the safety reference values set by EFSA, it can be concluded that the EPIC population was exposed to mycotoxins in a considered safe range (Table S2). The EU has its own established maximum limits (MSLs) for mycotoxins in foods and feeds, and it has the resources to strictly follow the crops from cultivation to storage [34]. Lee and Ryu (2017) collected data from publications since 2006 over a 10 year period to examine the presence of AFs in unprocessed food-grade grains (barley, maize, wheat, rice, and other cereals, such as oats) in Africa, the Americas, Asia, and Europe. This study found an overall AF prevalence in these regions of 55%, ranging from 15% in the Americas to 63% in Asia and for FB1, with total prevalence varying between 39% for Europe and 95% for America. The global DON prevalence was nearly 60%, whereby it varied from 50% in Asia to 76% Africa [10,35]. From this it can deduced that regional and cultural changes, climatic conditions, and the presence or absence of strict regulations influence the mycotoxin occurrence in food. Therefore, large occurrence and exposure data surveys on mycotoxin contamination should be performed across the world.

No statistically significant associations were found in the current study between the individual mycotoxins or mycotoxin groups and risk of RCC development (Table 3). Recently, a systematic review provided an overview of data linking exposure to different mycotoxins with human cancer risk. Only a few studies have investigated the associations between mycotoxin exposures and cancer risk. No articles investigating the relationship between mycotoxin exposure and the development of RCC were identified [15]. Therefore, the current investigation helps to close the research gap in linking common mycotoxin exposures to renal cancer risk.

### 4.2. Mycotoxins as Nephrotoxins?

Some mycotoxins are known to be natural nephrotoxins, more specifically, OTA, CIT, and FB1 [16]. These three have been proposed as associated with BEN [6,7,36]. Exposure to OTA and CIT was assessed in a Czech cohort of patients with kidney tumors [37,38]. The data indicated a frequent, but low dietary exposure to CIT and OTA, well below the respective health-based guidance values [8,39,40].

Although no association was observed in the current study, OTA is suggested to have carcinogenic effects on the kidneys through covalent DNA adduct formation, resulting in direct genotoxicity attributable to oxidative stress [12]. In an experiment with male Fischer rats fed with OTA, a kidney tumor was detected within the first 6 months, and the tumor incidence was raised by 25% [41]. This was because the genes in charge of kidney damage and cell regeneration were significantly affected by the action of OTA [42]. Next, CIT has been suspected to be one of the etiological agents of BEN and urinary tract tumors in humans [36,43]. CIT-specific DNA adducts were reported in the renal tumors of patients with BEN [44]. Molecular events underlying CIT-induced cell-cycle progression can explain the CIT-induced renal carcinogenesis [45]. Nevertheless, we were unable to evaluate CIT individually in relation to RCC because estimated CIT exposure was negligible in the EPIC cohort. Lastly, FB1 has been linked to the development of renal carcinomas in male rats [12,17,46]. It is well established that FB1-mediated disruption of sphingolipid metabolism plays a key role in FB1 toxicity [47].

Additionally, crops can be infected by different fungi; therefore, interactions between the produced mycotoxins can appear. These interactions can be synergistic, additive, or antagonistic [48]. For example, CIT can act together with OTA and interfere with RNA synthesis [12,49]. A number of studies have shown synergistic effects for endpoints related to the nephrotoxicity of OTA and CIT [48,50]. Further research should be undertaken to investigate the effect of co-exposure to mycotoxins on humans.

### 4.3. Strengths and Limitations of the Study

This investigation has many important strengths such as the unique access to one of the largest cohort databases currently available for investigating effects of dietary exposures on cancer risks. Strengths of the EPIC study include its large sample size, prospective design, longitudinal follow-up, and the inclusion of participants from different European countries with standardized data collection, especially for diet, which offers a broad perspective on dietary intakes in Europe. In addition, access to the EFSA mycotoxin occurrence data derived from the European member states and support and training given by the EFSA experts are additional imperative strengths. Correspondingly, the in-depth independent quality controls that were performed on various levels of these analyses underscores the quality of the results. To the authors’ knowledge, this is the first large-scale external exposure assessment in a prospective longitudinal cohort, where associations between mycotoxin exposure and the risk of developing RC were studied. Furthermore, it is the first large-scale study investigating the possible association in Europe.

However, some limitations of the study should be acknowledged, including the EPIC dietary intake assessment methods (self-reported dietary questionnaires) possibly being prone to reporting bias. Indeed, diet measurement instruments are built to capture the usual dietary intakes of an individual; however, they are still subject to imprecision and inaccuracy. In addition, only dietary intakes at baseline could be used since there were no dietary follow-up data available. Data obtained from detailed 24 h recalls collected in a subsample of the EPIC population (~30,000 participants) were used to analyze the recipes and complex foods included in the dietary questionnaires with the aim of standardizing and optimizing the quality of the dietary questionnaire data. Furthermore, there were insufficient cases to examine associations for different histological subtypes of RCC [51].

Moreover, the mycotoxin contamination levels in foods, reported in the EFSA occurrence database, importantly depend on the environmental factors such as climatic conditions, region, good agricultural practices, storage conditions, and food processing, leading to variations in mycotoxin concentrations measured in similar foods. Some countries (e.g., Germany) have more mycotoxin occurrence data in food in comparison with other. These findings may be somewhat limited since geographic matching of the EPIC data with the mycotoxin occurrence data from the specific European country was not possible due to the granularity of the EFSA database. Fungal spot contaminations can lead to heterogeneously distributed mycotoxin patterns over the samples. However, in epidemiological analyses, where individual data are being analyzed in a deterministic way (using point estimates), these variations cannot be taken into consideration. LOD and/or LOQ values in the EFSA occurrence database differed substantially as multiple analytical techniques were used to detect and quantify the mycotoxin concentration.

Caution is needed regarding the extrapolation of these results to the entire European population or to other populations or ethnicities worldwide since this study included volunteers from nine European countries involved in a longitudinal cohort study investigating the association between nutrition and health, with overall more health-conscious behaviors compared to the general population. Furthermore, in our models, we included all the participants with available dietary intake data, but with potential missing data on other covariates replaced with a ‘missing’ class or imputation. Although this may have induced some bias, a complete case model would lead to a selection toward more compliant participants in an already health-conscious population. Nevertheless, analyses with a complete case model provided similar results. Additionally, this study used a single assessment of dietary intakes at baseline. Although a consumer’s diet may change over time, it is usually hypothesized that this estimation reflects general eating behavior throughout middle-age adult life [52]. Lastly, this study was based on an observational cohort. Thus, even though our models included adjustment for a large range of confounding factors, residual confounding cannot be entirely ruled out.

### 4.4. Suggestions for Future Research, Further Perspectives, and Public Health Implications

This indirect approach, which is referred to as ‘external exposure’ estimation, gives the community a first insight into the global mycotoxin burden at the population level. The ‘internal exposure’ estimation by measuring biomarkers of exposure and effect in biological matrices takes into account additional variables such as mycotoxicokinetics and -dynamics. Therefore, additional information derived from biomarkers of exposure and effect is needed to characterize the physiological processes involved in any potential relationship between mycotoxin exposures and cancer risk. Investigating the internal exposure is essential to understand the human mycotoxicokinetics and to exclude the possible confounding issue of heterogeneous distribution of mycotoxins in foods. Hence, a complementary but more suitable and reliable mycotoxin exposure assessment can be achieved by the direct measurement of mycotoxin exposure biomarkers. Calculating intake levels from biomarker levels is still challenging; therefore, both external and internal exposure assessments are recommended when investigating and tackling health effects such as cancer risk of multiple mycotoxin exposures.

As mentioned before, there is a gap in cancer risk assessment relating to mycotoxin exposure. To date, most studies have been performed in the context of liver cancer [15]. However, mycotoxins may be relevant for many types of cancer [12]. Upcoming publications based on a similar study design and cohort show intriguing results with respect to hepatocellular carcinoma and colorectal cancer risk (Huybrechts et al., in submission) These investigations revealed positive significant associations of exposure to DON and fusarenon-X with hepatocellular carcinoma risk, while exposure to DON, PAT, and *Fusarium* toxins may increase colorectal cancer risk. Multi-mycotoxin exposures were associated with both hepatocellular and colorectal cancer development [53,54]. Yet, only few human epidemiological studies have investigated the associations between mycotoxin exposures and cancer risk [15]. Future studies on the current topic are, therefore, recommended.

The statistically nonsignificant results in this study may be due to the lack of sufficient occurrence data in the EFSA database of certain mycotoxins, such as CIT, FBs, and others. In addition, data on the occurrence of other emerging mycotoxins as nephrotoxins in food commodities are still too limited to reliably estimate human exposure [8,38]. Therefore, more occurrence data in different food commodities and countries are recommended to include in the EFSA database. This would enable a more reliable estimation with regard to mycotoxin exposures and possible associations with kidney cancer development. Furthermore, due to global warming, a transition is already taking place in which the toxigenic fungi that previously occurred in Africa can also be found in south and east Europe [55,56]. Therefore, future studies on the influence of climate change are recommended.

Moreover, some mycotoxins, such as enniatins and mycotoxins derived from *Alternaria*, have limited data. As a result, they have not been included in the IARC Monographs. This is due to sparse epidemiological, experimental, and/or mechanistic studies or due to non-carcinogenicity [57]. Significant new information might support a different classification. Recently, FB1 has been listed as a high priority for re-evaluation within 2.5 years, being a possible preventable cause of cancer [58].

In the past, studies have shown that exposure to multiple mycotoxins may result in interactions between the mycotoxins. For example, a synergistic effect was observed in kidneys of chickens after exposure to OTA and CIT [48,59]. Simultaneous exposure with OTA and ZEN resulted in an antagonistic effect on OTA-induced kidney damage [60]. Yet, not many other studies exist that examined the mechanistic effects of co-exposure. For this reason, further research should be undertaken to investigate the effect of co-exposure of mycotoxins on humans.

Investigating the internal exposure is essential. Consequently, additional information derived from biomarkers of exposure and effect are needed to characterize the physiological processes involved in any potential relationship between mycotoxin exposures and cancer risk. Calculating intake levels from biomarker levels is still challenging; therefore, both external exposure and internal exposure need to be considered when investigating and tackling health effects such as cancer risk of multiple mycotoxin exposures.

Cancer is an emerging health problem in low- and middle-income countries that needs to be addressed appropriately in order to control increased incidence and mortality rates [61,62,63]. Mycotoxin exposure levels might be considerably higher in certain regions due to climate and the absence of strict regulations. Recently, deaths have been documented in Kenya repeatedly from the consumption of aflatoxin-contaminated maize [64]. In addition, the greatest number of new cancer cases in 2020 was observed in Asia [61]. As a result, additional associations that are not observed in European cohorts, such as the lack of association seen for RCC herein, might be identified. Therefore, similar prospective cohorts in Africa, Asia, and other continents could help develop future public health strategies and make or adapt current mycotoxin legislation.

Lastly, it is clear that more evidence is needed to gain a more complete understanding of the etiology and risk factors for kidney cancer. Future research must, therefore, ensure an improved understanding of the underlying mechanisms of kidney cancer development. Molecular epidemiology, including but not limited to metabolomics and cancer genomics, is an exciting area of research that has yet to be fully used for the study of kidney cancer. Metabolomics can provide insight into metabolic derangements that underlie disease and lead to the discovery of new therapeutic treatments, as well as the discovery of biomarkers for early diagnosis and/or prognosis. Cancer genomics has the potential to causally link exogenous exposures or endogenous mechanisms to individual tumors via the identification of mutational signatures underlying these processes [51,65,66].

## 5. Conclusions

The results of this study did not show statistically significant associations between RCC risk and long-term dietary mycotoxin exposures. However, these results should be validated in other cohorts and preferably using repeated dietary exposure measurements. In addition, more occurrence data of CIT and FBs, in different food commodities and countries in the EFSA database, could help to provide further insights.

## Figures and Tables

**Table 1:** Characteristics of EPIC participants included in the analysis of mycotoxin exposures and risk of RCC.

RCC in EPIC				
	Non-Cases		RCC Cases	
Total sample after exclusions = 450,112	449,201		911	
	*Mean*	*SD*	*Mean*	*SD*
**Body mass index (kg/m^2^)**	25.3	4.2	27.0	4.3
**Age at recruitment (years)**	51.1	9.8	55.5	7.7
**Energy intake USDA (kcal/day)**	2076.3	618.7	2150.7	672.6
**Alcohol at recruitment (g/day)**	11.7	16.8	14.6	20.6
	*n*	*%*	*n*	*%*
**Sex**				
Male	130,931	29.1	495	54.3
Female	318,270	70.9	416	45.7
**Education**				
None	15,519	3.5	32	3.5
Primary school completed	110,722	24.6	342	37.5
Technical/professional school	103,564	23.1	219	24.0
Secondary school	93,787	20.9	123	13.5
Longer education (including university degree)	108,767	24.2	164	18.0
Not specified	16,842	3.7	31	3.4
**Physical activity**				
Inactive	87,829	19.6	203	22.3
Moderately inactive	149,613	33.3	328	36.0
Moderately active	120,001	26.7	198	21.7
Active	82,952	18.5	164	18.0
Missing	8806	2.0	18	2.0
**Diabetes**				
No	399,684	89.0	768	84.3
Yes	10,703	2.4	35	3.8
Do not know	38,814	8.6	108	11.9
**Hypertension**				
No	297,754	77.0	460	61.4
Yes	80,223	20.7	266	35.5
Do not know	8708	2.3	23	3.1
**Smoking status**				
Never	218,958	48.7	336	36.9
Former	122,399	27.2	281	30.8
Current	99,430	22.1	285	31.3
Unknown	8414	1.9	9	1.0

**Table 2:** Description of the external mycotoxin exposures assessed on the basis of dietary questionnaire data for the EPIC cohort. Table S1 shows the lower bound (LB) values, and Table S2 shows data relative to EFSA reference values.

Middle Bound (MB)—µg/kg Body Weight per Day										
LABEL (Expressed in µg/kg Body Weight/day)	Case Status	Mean	SD	Min	P05	P25	P50	P75	P95	Max
Ergot alkaloids (Middle bound-body weight-computed)	Non-case	0.07	0.07	0.00	0.01	0.03	0.05	0.09	0.19	1.73
	RCC case	0.07	0.06	0.00	0.01	0.03	0.06	0.10	0.20	0.43
Ochratoxins (Middle bound-body weight-computed)	Non-case	0.00	0.00	0.00	0.00	0.00	0.00	0.00	0.00	0.05
	RCC case	0.00	0.00	0.00	0.00	0.00	0.00	0.00	0.00	0.01
Aflatoxins (Middle bound-body weight-computed)	Non-case	0.00	0.00	0.00	0.00	0.00	0.00	0.00	0.01	0.03
	RCC case	0.00	0.00	0.00	0.00	0.00	0.00	0.00	0.01	0.01
Patulin (Middle bound-body weight)	Non-case	0.01	0.01	0.00	0.00	0.01	0.01	0.02	0.04	0.29
	RCC case	0.01	0.01	0.00	0.00	0.01	0.01	0.02	0.03	0.09
Deoxynivalenol and derivatives (Middle bound-body weight-computed)	Non-case	0.24	0.13	0.00	0.09	0.15	0.22	0.30	0.48	3.09
	RCC case	0.22	0.12	0.02	0.08	0.14	0.20	0.28	0.43	0.82
T-2/HT-2 toxins (Middle bound-body weight-computed)	Non-case	0.02	0.01	0.00	0.00	0.01	0.02	0.02	0.04	0.21
	RCC case	0.02	0.01	0.00	0.00	0.01	0.02	0.02	0.04	0.09
Nivalenol (Middle bound-body weight)	Non-case	0.03	0.02	0.00	0.01	0.02	0.03	0.04	0.07	0.35
	RCC case	0.03	0.02	0.00	0.01	0.02	0.03	0.04	0.06	0.13
Fumonisins (Middle bound-body weight-computed)	Non-case	0.24	0.13	0.00	0.09	0.15	0.21	0.30	0.48	2.71
	RCC case	0.21	0.12	0.02	0.08	0.14	0.19	0.26	0.44	1.49
Diacetoxyscirpenol (Middle bound -body weight)	Non-case	0.03	0.02	0.00	0.01	0.02	0.03	0.04	0.06	0.56
	RCC case	0.03	0.02	0.01	0.01	0.02	0.03	0.04	0.06	0.14
Zearalenone and derivatives (Middle bound-body weight-computed)	Non-case	0.04	0.02	0.00	0.01	0.02	0.03	0.04	0.08	0.71
	RCC case	0.03	0.03	0.01	0.01	0.02	0.03	0.04	0.07	0.42
Fusarium Toxins (Middle bound-body weight-computed)	Non-case	0.60	0.28	0.03	0.24	0.40	0.55	0.74	1.12	5.71
	RCC case	0.55	0.26	0.08	0.23	0.37	0.50	0.68	1.04	2.02
Fusarenon X (Middle bound-body weight)	Non-case	0.02	0.01	0.00	0.00	0.01	0.01	0.02	0.04	0.16
	RCC case	0.02	0.01	0.00	0.00	0.01	0.01	0.02	0.04	0.07
Sterigmatocystins (Middle bound-body weight)	Non-case	0.00	0.00	0.00	0.00	0.00	0.00	0.00	0.00	0.04
	RCC case	0.00	0.00	0.00	0.00	0.00	0.00	0.00	0.00	0.01
Moniliformine (Middle bound-body weight)	Non-case	0.00	0.01	0.00	0.00	0.00	0.00	0.00	0.01	0.44
	RCC case	0.00	0.01	0.00	0.00	0.00	0.00	0.00	0.02	0.10
Alternaria toxins (Middle bound-body weight-computed)	Non-case	0.19	0.10	0.00	0.05	0.12	0.18	0.25	0.39	1.36
	RCC case	0.19	0.10	0.02	0.05	0.12	0.18	0.25	0.36	0.78
Citrinin (Middle bound-body weight)	Non-case	0.00	0.00	0.00	0.00	0.00	0.00	0.00	0.00	0.00
	RCC case	0.00	0.00	0.00	0.00	0.00	0.00	0.00	0.00	0.00
Beauvericin (Middle bound-body weight)	Non-case	0.00	0.00	0.00	0.00	0.00	0.00	0.00	0.00	0.00
	RCC case	0.00	0.00	0.00	0.00	0.00	0.00	0.00	0.00	0.00
Enniatins (Middle bound-body weight-computed)	Non-case	0.05	0.05	0.00	0.00	0.01	0.03	0.06	0.15	0.97
	RCC case	0.05	0.05	0.00	0.00	0.01	0.03	0.07	0.14	0.38
Total mycotoxins (Middle bound-body weight-computed)	Non-case	0.93	0.43	0.06	0.38	0.62	0.86	1.16	1.74	6.55
	RCC case	0.89	0.42	0.14	0.37	0.60	0.82	1.09	1.67	2.96

Abbreviations: European Prospective Investigation into Cancer and Nutrition (EPIC) cohort, Lower bound (LB), middle bound (MB), renal cell carcinoma (RCC). The variables in bold show that a large part of the EPIC population, regardless of case status, is exposed to some of the main mycotoxins present in European foods such as DON and derivatives, fumonisins, Fusarium toxins, Alternaria toxins, and total mycotoxins.

**Table 3:** Hazard ratios (HRs) and their 95% confidence intervals (CIs) for the associations between mycotoxin exposures (μg/body weight/day) and renal cell carcinoma risk using a fully adjusted model * for the continuous exposure and tertiles (*n* = 450,112; cases = 911 and non-cases = 449,201). The HRs for the continuous variables have been computed on the log_2_ scale: risk for a doubling of the exposure. Tests for trend were computed using the tertile score (1–3 as a continuous variable) specific to each mycotoxin.

Middle-Bound Scenario (MB)											
Mycotoxins (µg/kg bw/day)	Cases *N* = 911	Hazard Ratio	95% Confidence Interval	ProbChiSq (P)	TrendTest	Mycotoxins (µg/kg bw/day)	Cases *N* = 911	Hazard Ratio	95% Confidence Interval	ProbChiSq (P)	TrendTest
**15-Acetyl-deoxynivalenol**	911	0.94	0.81–1.10	0.4309		**3-Acetyl-deoxynivalenol**	911	0.94	0.81–1.09	0.4031	
T1	334	1	Ref.			T1	326	1	Ref.		
T2	308	1.05	0.86–1.29	0.6254	0.6169	T2	314	1.06	0.86–1.29	0.6000	0.5004
T3	269	1.06	0.84–1.35	0.6147		T3	271	1.09	0.85–1.38	0.4974	
											
**Aflatoxin B1**	911	0.96	0.82–1.14	0.6590		**Aflatoxin B2**	911	0.97	0.83–1.12	0.6484	
T1	404	1	Ref.	.		T1	391	1	Ref.		
T2	278	0.88	0.72–1.07	0.1949	0.1473	T2	298	0.97	0.80–1.17	0.7336	0.1735
T3	229	0.84	0.65–1.07	0.1615		T3	222	0.84	0.65–1.07	0.1579	
											
**Aflatoxin G1**	911	0.97	0.83–1.13	0.6962		**Aflatoxin G2**	911	0.95	0.82–1.11	0.5270	
T1	395	1	Ref.			T1	394	1	Ref.		
T2	295	0.96	0.79–1.16	0.6727	0.1979	T2	301	0.96	0.79–1.16	0.6841	0.1370
T3	221	0.85	0.66–1.08	0.1849		T3	216	0.82	0.64–1.05	0.1207	
											
**Aflatoxin M1**	911	0.98	0.93–1.03	0.4426		**Aflatoxin (sum of B1. B2. G1. G2)**	911	1.08	0.94–1.24	0.2913	
T1	351	1	Ref.			T1	378	1	Ref.		
T2	311	1.02	0.84–1.23	0.8588	0.5349	T2	284	1.01	0.83–1.23	0.9086	0.2293
T3	249	0.93	0.74–1.16	0.5034		T3	249	1.16	0.91–1.48	0.2176	
											
**Altenuene**	911	1.06	0.92–1.22	0.4326		**Alternaria alternata F. sp. lycopersici toxins**	911	0.98	0.88–1.08	0.6385	
T1	320	1	Ref.			T1	320	1	Ref.		
T2	318	1.06	0.87–1.31	0.5514	0.7268	T2	318	1.01	0.82–1.23	0.9440	0.8915
T3	273	0.96	0.75–1.24	0.7591		T3	273	0.98	0.77–1.25	0.8919	
											
**Altertoxin I**	911	1.00	0.90–1.13	0.9427		**Alternariol monomethyl ether**	911	1.01	0.94–1.09	0.8195	
T1	314	1	Ref.			T1	306	1	Ref.		
T2	305	1.01	0.82–1.24	0.9257	0.6751	T2	309	1.12	0.91–1.37	0.2909	0.4432
T3	292	0.95	0.76–1.21	0.6984		T3	296	1.10	0.86–1.42	0.4417	
											
**Alternariol**	911	1.03	0.91–1.17	0.6629		**Deoxynivalenol-3-glucoside**	911	1.01	0.97–1.05	0.6683	
T1	312	1	Ref.			T1	344	1	Ref.		
T2	311	1.00	0.81–1.24	0.9919	0.9257	T2	298	1.06	0.89–1.28	0.5060	0.5181
T3	288	1.01	0.78–1.32	0.9269		T3	269	1.07	0.85–1.34	0.5836	
											
**Deoxynivalenol**	911	0.97	0.84–1.14	0.7357		**Enniatin A1**	911	1.04	0.97–1.10	0.2474	
T1	360	1	Ref.			T1	316	1	Ref.		
T2	307	0.93	0.76–1.14	0.4626	0.5667	T2	294	0.96	0.77–1.20	0.7215	0.6437
T3	244	0.93	0.71–1.21	0.5820		T3	301	0.93	0.70–1.25	0.6437	
											
**Enniatin A**	911	1.00	0.96–1.04	0.9364		**Enniatin B1**	911	1.03	0.98–1.10	0.2441	
T1	307	1	Ref.			T1	318	1	Ref.		
T2	325	1.02	0.84–1.23	0.8748	0.1160	T2	293	1.00	0.80–1.25	0.9799	0.9896
T3	279	0.82	0.64–1.04	0.0947		T3	300	1.00	0.75–1.34	0.9905	
											
**Enniatin B**	911	1.02	0.97–1.07	0.4636		**Ergocorninine**	911	1.03	0.96–1.11	0.4088	
T1	322	1	Ref.			T1	307	1	Ref.		
T2	284	0.98	0.78–1.23	0.8910	0.8307	T2	285	0.87	0.69–1.09	0.2194	0.8448
T3	305	0.97	0.72–1.30	0.8309		T3	319	1.00	0.77–1.31	0.9880	
											
**Ergocornine**	911	1.03	0.95–1.11	0.4684		**Ergocristinine**	911	1.03	0.96–1.11	0.4098	
T1	305	1	Ref.			T1	305	1	Ref.		
T2	283	0.88	0.70–1.11	0.2828	0.4757	T2	278	0.85	0.68–1.06	0.1560	0.3873
T3	323	1.08	0.82–1.41	0.5947		T3	328	1.09	0.84–1.42	0.5301	
											
**Ergocristine**	911	1.04	0.97–1.11	0.2354		**alpha-Ergocryptinine**	911	1.01	0.94–1.08	0.7996	
T1	297	1	Ref.			T1	293	1	Ref.		
T2	307	1.08	0.85–1.38	0.5228	0.1475	T2	289	0.94	0.75–1.18	0.6148	0.2091
T3	307	1.22	0.92–1.62	0.1675		T3	329	1.16	0.89–1.52	0.2680	
											
**alpha-Ergocryptine**	911	1.01	0.94–1.08	0.8209		**beta-Ergocryptine**	911	0.99	0.97–1.02	0.6153	
T1	304	1	Ref.			T1	287	1	Ref.		
T2	279	0.87	0.70–1.09	0.2316	0.6508	T2	279	1.04	0.78–1.38	0.8019	0.0298
T3	328	1.06	0.81–1.40	0.6611		T3	345	1.34	0.98–1.82	0.0657	
											
**Ergocryptine (alpha + beta epimers)**	911	0.98	0.96–1.01	0.1971		**Ergometrinine**	911	1.01	0.94–1.07	0.8427	
T1	272	1	Ref.			T1	300	1	Ref.		
T2	282	1.01	0.78–1.31	0.9214	0.6220	T2	293	0.92	0.74–1.15	0.4681	0.3190
T3	357	1.07	0.79–1.46	0.6531		T3	318	1.14	0.87–1.48	0.3370	
											
**Ergometrine**	911	1.03	0.97–1.10	0.3151		**Ergosine**	911	1.02	0.95–1.10	0.5265	
T1	295	1	Ref.			T1	305	1	Ref.		
T2	302	0.99	0.79–1.24	0.9372	0.5369	T2	296	0.96	0.77–1.20	0.7302	0.8886
T3	314	1.08	0.83–1.40	0.5762		T3	310	1.02	0.77–1.35	0.8739	
											
**Ergosinine**	911	1.01	0.95–1.06	0.8357		**Ergotaminine**	911	1.01	0.94–1.08	0.7761	
T1	286	1	Ref.			T1	301	1	Ref.		
T2	297	1.00	0.80–1.25	0.9787	0.1321	T2	277	0.89	0.71–1.12	0.3207	0.5503
T3	328	1.21	0.93–1.58	0.1616		T3	333	1.07	0.82–1.41	0.6168	
											
**Ergotamine**	911	1.02	0.96–1.08	0.4818		**Fumonisin B1**	911	0.98	0.86–1.12	0.7649	
T1	297	1	Ref.			T1	397	1	Ref.		
T2	292	0.88	0.70–1.12	0.3072	0.9440	T2	298	0.88	0.72–1.08	0.2155	0.1050
T3	322	1.02	0.76–1.37	0.9034		T3	216	0.81	0.63–1.05	0.1083	
											
**Fumonisin B2**	911	0.90	0.76–1.07	0.2239		**Fumonisin B3**	911	0.99	0.93–1.07	0.8851	
T1	393	1	Ref.			T1	356	1	Ref.		
T2	290	0.81	0.67–0.99	0.0443	0.0577	T2	303	1.05	0.86–1.27	0.6473	0.2159
T3	228	0.78	0.60–1.02	0.0647		T3	252	1.15	0.92–1.44	0.2098	
											
**Fumonisins**	911	0.95	0.87–1.03	0.2415		**HT-2 toxin**	911	0.94	0.85–1.03	0.1996	
T1	361	1	Ref.			T1	339	1	Ref.		
T2	287	0.95	0.78–1.15	0.5829	0.3191	T2	301	0.95	0.78–1.15	0.6069	0.8375
T3	263	0.89	0.71–1.12	0.3189		T3	271	0.98	0.79–1.21	0.8392	
											
**Ochratoxin A**	911	1.05	0.91–1.21	0.5121		**Sum T-2 and HT-2**	911	0.94	0.82–1.07	0.3462	
T1	335	1	Ref.			T1	357	1	Ref.		
T2	316	1.12	0.92–1.37	0.2532	0.3719	T2	281	0.89	0.73–1.09	0.2760	0.3180
T3	260	1.12	0.88–1.42	0.3617		T3	273	0.89	0.70–1.12	0.3074	
											
**Tentoxin**	911	1.04	0.92–1.17	0.5588		**Tenuazonic acid**	911	0.98	0.85–1.12	0.7559	
T1	304	1	Ref.			T1	320	1	Ref.		
T2	308	1.03	0.84–1.26	0.7930	0.6612	T2	302	0.94	0.77–1.14	0.5244	0.3046
T3	299	0.95	0.75–1.21	0.7000		T3	289	0.89	0.70–1.12	0.3039	
											
**T-2 toxin**	911	0.93	0.84–1.04	0.2288		**Zearalanone**	911	0.99	0.96–1.02	0.5669	
T1	351	1	Ref.			T1	289	1	Ref.		
T2	287	0.89	0.73–1.09	0.2771	0.9064	T2	331	1.04	0.85–1.27	0.7089	0.6126
T3	273	0.98	0.78–1.24	0.8935		T3	291	0.95	0.77–1.18	0.6585	
											
**alpha-Zearalenol**	911	1.02	0.96–1.08	0.4780		**beta-Zearalenol**	911	1.02	0.96–1.09	0.5133	
T1	356	1	Ref.			T1	362	1	Ref.		
T2	274	0.96	0.80–1.15	0.6592	0.5857	T2	281	0.96	0.80–1.16	0.6861	0.8140
T3	281	1.09	0.87–1.37	0.4523		T3	268	0.98	0.78–1.23	0.8751	
											
**Zearalenol**	911	0.96	0.89–1.03	0.2565		**Zearalenone**	911	0.98	0.86–1.11	0.7149	
T1	338	1	Ref.			T1	408	1	Ref.		
T2	317	0.90	0.74–1.10	0.3135	0.2477	T2	257	0.92	0.75–1.12	0.3968	0.6439
T3	256	0.88	0.70–1.09	0.2428		T3	246	0.95	0.74–1.22	0.6863	

**Ergot alkaloids**	911	1.04	0.97–1.11	0.2728		**Ochratoxins**	911	1.05	0.91–1.22	0.4987	
T1	298	1	Ref.			T1	336	1	Ref.		
T2	283	0.87	0.68–1.10	0.2413	0.4738	T2	309	1.11	0.91–1.35	0.3177	0.3153
T3	330	1.08	0.82–1.42	0.6033		T3	266	1.13	0.89–1.43	0.3092	
											
**Aflatoxins**	911	1.00	0.84–1.19	0.9790		**Patulin**	911	0.99	0.92–1.06	0.7407	
T1	383	1	Ref.			T1	354	1	Ref.		
T2	287	0.96	0.78–1.16	0.6485	0.7878	T2	288	0.94	0.78–1.13	0.5041	0.9347
T3	241	0.97	0.75–1.24	0.8041		T3	269	1.00	0.81–1.22	0.9628	
											
**Deoxynivalenol and derivatives**	911	0.96	0.81–1.14	0.6726		**T-2/HT-2 toxins**	911	0.94	0.85–1.04	0.2117	
T1	364	1	Ref.			T1	350	1	Ref.		
T2	308	0.99	0.80–1.21	0.8913	0.4282	T2	297	0.92	0.75–1.11	0.3720	0.4750
T3	239	0.89	0.68–1.17	0.4169		T3	264	0.92	0.74–1.15	0.4750	
											
**Nivalenol**	911	0.96	0.86–1.08	0.5140		**Fumonisins**	911	0.95	0.82–1.11	0.5282	
T1	335	1	Ref.			T1	382	1	Ref.		
T2	304	0.97	0.79–1.19	0.7938	0.5432	T2	305	1.02	0.84–1.24	0.8419	0.4525
T3	272	0.92	0.72–1.19	0.5425		T3	224	0.91	0.71–1.16	0.4338	
											
**Diacetoxyscirpenol**	911	0.96	0.82–1.12	0.5779		**Zearalenone and derivatives**	911	0.94	0.82–1.09	0.4176	
T1	373	1	Ref.			T1	403	1	Ref.		
T2	304	0.91	0.75–1.11	0.3767	0.3009	T2	271	0.96	0.79–1.17	0.7036	0.2206
T3	234	0.88	0.68–1.13	0.3123		T3	237	0.85	0.66–1.09	0.2071	
											
**Fusarium toxins**	911	0.93	0.77–1.13	0.4961		**Fusarenon X**	911	1.04	0.94–1.14	0.4467	
T1	382	1	Ref.			T1	322	1	Ref.		
T2	291	0.94	0.77–1.15	0.5631	0.5219	T2	318	1.08	0.88–1.32	0.4499	0.6512
T3	238	0.92	0.71–1.20	0.5288		T3	271	1.06	0.82–1.36	0.6659	
											
**Sterigmatocystins**	911	0.98	0.94–1.02	0.3215		**Moniliformine**	911	1.00	0.95–1.02	0.7841	
T1	386	1	Ref.			T1	332	1	Ref.		
T2	297	0.94	0.79–1.12	0.4939	0.0907	T2	302	0.96	0.80–1.17	0.7097	0.8612
T3	228	0.82	0.66–1.03	0.0829		T3	277	1.02	0.83–1.26	0.8238	
											
**Alternaria toxins**	911	1.05	0.89–1.25	0.5417		**Citrinin**	911	1.00	0.96–1.03	0.8126	
T1	310	1	Ref.			T1	327	1	Ref.		
T2	313	1.12	0.90–1.38	0.3119	0.7951	T2	316	1.06	0.87–1.28	0.5694	0.9187
T3	288	1.04	0.80–1.37	0.7497		T3	268	0.99	0.80–1.21	0.8992	
											
**Enniatins**	911	1.02	0.97–1.08	0.4021		**All Mycotoxins**	911	0.99	0.81–1.22	0.9424	
T1	307	1	Ref.			T1	370	1	Ref.		
T2	305	0.92	0.74–1.13	0.4071	0.3679	T2	290	0.85	0.69–1.05	0.1375	0.1313
T3	299	0.88	0.67–1.16	0.3813		T3	251	0.81	0.62–1.07	0.1366	

(*) Fully adjusted model: stratified by sex, study center, and age at recruitment, and adjusted for BMI, energy intake, alcohol at recruitment, sex, education, physical activity index, diabetes, hypertension, and smoking status. Abbreviations: body weight (bw), 95% confidence interval (CI), first tertile is the reference tertile (T1, Ref.), hazard ratio (HR), middle bound (MB), second tertile (T2), third tertile (T3). For test of linear trends across tertiles, participants were assigned the score (1 to 3) of each category, and the corresponding variable was modeled as a continuous term. All *p*-values between the tertiles, known as TrendTest, and all *p*-values comparing each tertile with the reference, known as ProbChiSq, were below 0.05. Therefore, no statistically significant associations were found between any studied mycotoxin or group and risk of RCC development. Moreover, the multi-adjusted Cox hazard models did not result in significant associations between the individual mycotoxin exposures and RCC risk.

## Data Availability

EPIC data and biospecimens are available for investigators who seek to answer important questions on health and disease in the context of research projects that are consistent with the legal and ethical standard practices of IARC/WHO and the EPIC Centers. The primary responsibility for accessing the data obtained in the frame of the present publication belongs to the EPIC centers that provided them. The use of a random sample of anonymized data from the EPIC study can be requested by contacting epic@iarc.fr. The request will then be passed on to members of the EPIC Steering Committee for deliberation.

## Supplementary Materials

The following supporting information can be downloaded at https://www.mdpi.com/article/10.3390/nu14173581/s1, Table S1: Description of the external mycotoxin exposures assessed on the basis of dietary questionnaire data for the EPIC cohort; Table S2: Percentage (%) of the EPIC population with external mycotoxin exposures assessed on the basis of dietary questionnaire data below and above the safety reference values set by EFSA. References [37,67,68,69,70,71,72] are cited in the Supplementary Materials.
