# Supplementary material for: Mycotoxin Exposure and Renal Cell Carcinoma Risk: An Association Study in the EPIC European Cohort
Source: Nutrients. 2022 Aug 30;14(17):3581. doi: 10.3390/nu14173581 (PMC9460795; doi:10.3390/nu14173581)
Supplement: Supplementary file 1 [file nutrients-14-03581-s001.zip › nutrients-1861719-supplementary-revised.pdf]

nutrients

MDPI

Table S1. Description of the external mycotoxin exposures assessed based upon dietary questionnaire data for the EPIC cohort. (Table S1: lower bound values) (Table 2: middle bound values, can be found in the article)

Table S1 Lower Bound (LB) - µg/kg body weight per day										
**LABEL (expressed in µg/kg body weight/d)**	**Case status**	**Mean**	**Std**	**Min**	**P05**	**P25**	**P50**	**P75**	**P95**	**Max**
Ergot alkaloids (Lower bound - body weight - computed)	Non-case	0.02	0.03	0.00	0.00	0.00	0.01	0.02	0.07	1.57
	RCC case	0.02	0.03	0.00	0.00	0.00	0.01	0.02	0.08	0.36
Ochratoxins (Lower bound - body weight - computed)	Non-case	0.00	0.00	0.00	0.00	0.00	0.00	0.00	0.00	0.05
	RCC case	0.00	0.00	0.00	0.00	0.00	0.00	0.00	0.00	0.01
Aflatoxins (Lower bound - body weight - computed)	Non-case	0.00	0.00	0.00	0.00	0.00	0.00	0.00	0.00	0.01
	RCC case	0.00	0.00	0.00	0.00	0.00	0.00	0.00	0.00	0.00
Patulin (Lower bound - body weight)	Non-case	0.00	0.01	0.00	0.00	0.00	0.00	0.00	0.01	0.21
	RCC case	0.00	0.01	0.00	0.00	0.00	0.00	0.00	0.01	0.08
**Deoxynivalenol and derivatives** (Lower bound - body weight - computed)	Non-case	0.08	0.07	0.00	0.01	0.02	0.06	0.10	0.21	1.35
	RCC case	0.07	0.06	0.00	0.01	0.02	0.05	0.10	0.18	0.40
T-2/HT-2 toxins (Lower bound - body weight - computed)	Non-case	0.00	0.00	0.00	0.00	0.00	0.00	0.00	0.00	0.17
	RCC case	0.00	0.00	0.00	0.00	0.00	0.00	0.00	0.01	0.03
Nivalenol (Lower bound - body weight)	Non-case	0.00	0.00	0.00	0.00	0.00	0.00	0.00	0.00	0.01
	RCC case	0.00	0.00	0.00	0.00	0.00	0.00	0.00	0.00	0.00
**Fumonisins** (Lower bound - body weight - computed)	Non-case	0.05	0.06	0.00	0.00	0.02	0.03	0.06	0.13	2.42
	RCC case	0.05	0.08	0.00	0.00	0.01	0.03	0.05	0.13	1.18
Diacetoxyscirpenol (Lower bound -body weight)	Non-case	0.00	0.00	0.00	0.00	0.00	0.00	0.00	0.00	0.00
	RCC case	0.00	0.00	0.00	0.00	0.00	0.00	0.00	0.00	0.00
Zearalenone & derivatives (Lower bound - body weight - computed)	Non-case	0.01	0.02	0.00	0.00	0.00	0.01	0.01	0.04	0.69
	RCC case	0.01	0.02	0.00	0.00	0.00	0.01	0.01	0.05	0.40
	Non-case	0.14	0.10	0.00	0.04	0.07	0.11	0.17	0.32	2.53

**Fusarium Toxins** (Lower bound - body weight - computed)	RCC case	0.13	0.10	0.01	0.03	0.07	0.11	0.16	0.31	1.33
Fusarenon X	Non-case	0.00	0.00	0.00	0.00	0.00	0.00	0.00	0.00	0.00
(Lower bound - body weight)	RCC case	0.00	0.00	0.00	0.00	0.00	0.00	0.00	0.00	0.00
Sterigmatocystins	Non-case	0.00	0.00	0.00	0.00	0.00	0.00	0.00	0.00	0.00
(Lower bound - body weight)	RCC case	0.00	0.00	0.00	0.00	0.00	0.00	0.00	0.00	0.00
Moniliformine	Non-case	0.00	0.01	0.00	0.00	0.00	0.00	0.00	0.01	0.44
(Lower bound - body weight)	RCC case	0.00	0.01	0.00	0.00	0.00	0.00	0.00	0.01	0.09
**Alternaria toxins**	Non-case	0.01	0.01	0.00	0.00	0.00	0.00	0.01	0.02	0.40
(Lower bound - body weight - computed)	RCC case	0.01	0.01	0.00	0.00	0.00	0.00	0.01	0.03	0.11
Citrinin	Non-case	0.00	0.00	0.00	0.00	0.00	0.00	0.00	0.00	0.00
(Lower bound - body weight)	RCC case	0.00	0.00	0.00	0.00	0.00	0.00	0.00	0.00	0.00
Beauvericin	Non-case	0.00	0.00	0.00	0.00	0.00	0.00	0.00	0.00	0.00
(Lower bound - body weight)	RCC case	0.00	0.00	0.00	0.00	0.00	0.00	0.00	0.00	0.00
Enniatins	Non-case	0.05	0.05	0.00	0.00	0.01	0.03	0.06	0.15	0.96
(Lower bound - body weight - computed)	RCC case	0.05	0.05	0.00	0.00	0.01	0.03	0.07	0.14	0.37
**Total mycotoxins**	Non-case	0.21	0.14	0.00	0.06	0.11	0.18	0.27	0.48	3.11
(Lower bound - body weight - computed)	RCC case	0.21	0.15	0.01	0.05	0.11	0.17	0.26	0.46	1.72

Abbreviations: European Prospective Investigation into Cancer and Nutrition (EPIC), Lower Bound (LB), Middle Bound (MB), Renal Cell Carcinoma (RCC). The variables in bold show that a large part of the EPIC population is exposed to some of the main mycotoxins present in European foods such as *DON and derivatives*, *fumonisins*, *Fusarium* toxins, *Alternaria* toxins and total mycotoxins.

Table S2. Percentage (%) of the EPIC population with external mycotoxin exposures assessed based upon dietary questionnaire data below and above the safety reference values set by EFSA.

N = 450,112	Type	Cut-off	Reference	Lower Bound				Middle Bound			
LABEL (expressed in µg/kg body weight)				N ≤ cut-off	%	N > cut-off	%	N ≤ cut-off	%	N > cut-off	%
Ergot alkaloids	TDI	0.6	[67]	450,103	99.998	9	0.002	450,012	99.978	100	0.022
	ARfD	1		450,112	100	.	.	450,108	99.999	4	0.001
Ochratoxins sum	TDI	0.01714	[37]	450,025	99.981	87	0.019	449,985	99.972	127	0.028
Aflatoxin sum	BMDL	0.4	[68]	450,112	100	.	.	450,112	100	.	.
Patulin	PMTDI	0.4	[69]	450,112	100	.	.	450,112	100	.	.
Deoxynivalenol and derivatives	PMTDI	1	[70]	450,101	99.998	11	0.002	449,786	99.928	326	0.072
T-2/HT-2 toxins sum	TDI	0.1	[71]	450,112	100	.	.	450,103	99.998	9	0.002
Fumonisins sum	PMTDI	2	[70]	450,109	99.999	3	0.001	450,109	99.999	3	0.001
Zearalenone and derivatives sum	TDI	0.25	[72]	449,926	99.959	186	0.041	449,761	99.922	351	0.078

Abbreviations: Acute Reference Dose (ARfD), BenchMark Dose (BMD), Lower confidence bound of BMD (BMDL), European Food Safety Authority (EFSA), European Prospective Investigation into Cancer and Nutrition (EPIC), Lower Bound (LB), Middle Bound (MB), Provisional Maximum Tolerable Daily Intake (PMTDI), Tolerable Daily Intake (TDI)
